# VEGF-Production by CCR2-Dependent Macrophages Contributes to Laser-Induced Choroidal Neovascularization

**DOI:** 10.1371/journal.pone.0094313

**Published:** 2014-04-08

**Authors:** Torsten A. Krause, Anne F. Alex, Daniel R. Engel, Christian Kurts, Nicole Eter

**Affiliations:** 1 Institute of Experimental Immunology, Rheinische Friedrich-Wilhelms-University, Bonn, Germany; 2 Department of Ophthalmology, University of Münster, Münster, Germany; University Medical Center of the Johannes Gutenberg University of Mainz, Germany

## Abstract

Age-related macular degeneration (AMD) is the most prevalent cause of blindness in the elderly, and its exsudative subtype critically depends on local production of vascular endothelial growth factor A (VEGF). Mononuclear phagocytes, such as macrophages and microglia cells, can produce VEGF. Their precursors, for example monocytes, can be recruited to sites of inflammation by the chemokine receptor CCR2, and this has been proposed to be important in AMD. To investigate the role of macrophages and CCR2 in AMD, we studied intracellular VEGF content in a laser-induced murine model of choroidal neovascularisation. To this end, we established a technique to quantify the VEGF content in cell subsets from the laser-treated retina and choroid separately. 3 days after laser, macrophage numbers and their VEGF content were substantially elevated in the choroid. Macrophage accumulation was CCR2-dependent, indicating recruitment from the circulation. In the retina, microglia cells were the main VEGF^+^ phagocyte type. A greater proportion of microglia cells contained VEGF after laser, and this was CCR2-independent. On day 6, VEGF-expressing macrophage numbers had already declined, whereas numbers of VEGF^+^ microglia cells remained increased. Other sources of VEGF detectable by flow cytometry included in dendritic cells and endothelial cells in both retina and choroid, and Müller cells/astrocytes in the retina. However, their VEGF content was not increased after laser. When we analyzed flatmounts of laser-treated eyes, CCR2-deficient mice showed reduced neovascular areas after 2 weeks, but this difference was not evident 3 weeks after laser. In summary, CCR2-dependent influx of macrophages causes a transient VEGF increase in the choroid. However, macrophages augmented choroidal neovascularization only initially, presumably because VEGF production by CCR2-independent eye cells prevailed at later time points. These findings identify macrophages as a relevant source of VEGF in laser-induced choroidal neovascularization but suggest that the therapeutic efficacy of CCR2-inhibition might be limited.

## Introduction

Age-related macular degeneration (AMD) [1] is the most prevalent cause of blindness of the elderly [2], [3]. The rapidly progressing form of AMD is characterized by choroidal neovascularisation (CNV), i.e. the growth of new blood vessels, mainly from the choroid through Bruch's membrane [1]. The vessels can extend through the retinal pigment epithelium (RPE) into the neuroretina and tend to leak, thereby causing edema and bleeding and eventually the destruction of the photoreceptor cells with loss of vision in the central field.

Typical features of exsudative AMD, such as neovascularization and inflammatory phagocyte recruitment, can be analyzed in a mouse model by placing laser-spots with a standard thermal laser. The laser destroys the blood-retina barrier. This leads to recruitment of immune cells from the circulation, in particular phagocytes like macrophages (MP), dendritic cells (DCs) and neutrophils [4]. Microglia cells (μGlia), the resident phagocytes in the inner retina, translocate into the subretinal space and accumulate near the retinal pigment epithelium, which is also observed in human AMD [5]. Prior to these changes in exudative AMD an accumulation of lipoproteinacious deposits called drusen appears. Human drusenoid material contains proteins of MP/μGlia origin, such as the chemokine receptor CX_3_CR1 and the major histocompatibility complex molecules [5], [6], supporting the idea that mononuclear phagocytes contribute to the formation of drusen. On the other hand, there is evidence that macrophages play a protective role in CNV formation, e.g. by removing drusen material [7]. However, the role of MPs in AMD is not fully understood at present.

Neovascular AMD is primarily associated with the increased expression of the vascular endothelial growth factor A (VEGF), a potent angiogenic factor [8]. VEGF inhibition is a highly effective treatment in AMD [9]. MPs can produce VEGF in response to laser injury [10], [11], suggesting that these cells might promote CNV directly. In support of this, depleting these cells by clodronate liposomes diminished the size of neovascular areas in CNV [12]. However, the side effects resulting from systemic phagocyte depletion preclude this manoeuvre for therapeutic purposes in human AMD.

The recruitment and migration of immune cells is controlled by chemokines. The chemokine receptor CCR2 and its ligand CCL2 are especially important for migration of mononuclear phagocytes [13], [14]. CCL2 production was increased after laser injury [10] and in CX_3_CR1-deficient mice, resulting in increased influx of neurotoxic MPs that promoted photoreceptor degeneration [15]. Further increased CCL2 levels were also detected in human atrophic AMD [15]. Inhibiting CCR2/CCL2 signal by a CCR2 antagonist or by antibodies against CCL2 reduced MP recruitment, VEGF levels in the RPE-choroid complex, and the extent of laser-induced CNV size [10], [16]. Consistently CCR2^-/-^ mice showed reduced CNV [17]. Based on these findings, it was suggested that aspects of human AMD might be treated by CCR2 inhibition [15], [16]. This theoretically should inhibit MP recruitment and reduce VEGF production from these cells, while avoiding the side effects resulting from systemic MP depletion.

In addition to recruited MP, many resident eye cells have been reported to produce VEGF, including retinal astrocytes [18], [19], Müller cells under hypoxic conditions [20], [21] and the RPE [22]. RPE cells cultured with activated μGlia produce pro-inflammatory, chemotactic and pro-angiogenic molecules [23], including CCL2 and VEGF. The quantitative contribution of different cell types of the eye to intraocular VEGF production and its impact on CNV is unknown.

To answer these questions, we used a murine laser-induced CNV model and analysed VEGF production by flow cytometry. This technique allows simultaneous co-staining for several cell type markers, allowing to classify VEGF-producing cells, as we have reported previously [4]. Furthermore, we employed CCR2-deficient mice to clarify which of the VEGF-producing cells of the eye are dependent on this chemokine receptor. We here report that only one cell type in the eye, namely MP, upregulate VEGF after laser injury in a CCR2-dependent manner. However, their influence on CNV is only transient, arguing against the use of CCR2 inhibitors in AMD.

## Methods

### Animals

All animal experiments had been approved by a governmental ethics board at the ministry of nature, environment and consumer protection of the German state of Northrhine Westphalia (LANUV Recklinghausen permit number 8.87-50.10.35.08.326). Laser treatment was performed under anesthesia as described below. All efforts were made to minimize suffering. All mice were bred and kept under Specific Pathogen Free (SPF) conditions at the central animal facilities of Bonn and Münster university clinic. Mice had been backcrossed >10 times to the C57BL/6 background.

### Laser-induced CNV model

Eyes were treated with an argon green laser with a laser spot size set to 50 μm, duration of 0.1 seconds and an energy of 200 mW. Therefore, the mice were anaesthetized by isofluran inhalation.

The pupils were dilated with Neosynephrin-POS® 5% and Cyclopentolat Alcon® 1% eye drops. Additionally the cornea was locally anesthetized with Proparakaine® 1%. Then the mice were hold in front of the slit lamp and their eyes kept behind a cover glass spread with Methocel to focus on the fundus of the eye. Successfull laser treatment with rupturing the RPE and Bruch's membrane was determined by optical confirmation of typical bubbles on the apical site of the RPE-choroidal tissue.

### Fluorescence Microscopy of Choroidal Flatmounts and Quantification of CNV areas

The mice were sacrificed 2 or 3 weeks after laser injury by anaesthesia and cervical dislocation. The eyes were enucleated and adherent tissues removed. Fixation was done in 4% PFA (PBS) for one hour. Retinal and choroidal tissues were separated and washed in PBS (ICC-buffer) containing 1% BSA (PAA), 0.2% Tween20 (AppliChem) and 0.1% Triton-X 100 (Sigma). To visualize the endothelial cells (ECs) in neovascular areas, tissues were incubated in ICC-buffer with Alexa Fluor® 568-conjugated isolectin GS-IB4 (IB4) from *Griffonia simplicifolia* (life technologies) for 4h at 4°C. Then they were washed several times in ICC-buffer and mounted in Immumount on glass slides. The flatmounts were analyzed with an IX71 fluorescence microscope (Olympus) at 20-fold magnification. The size of IB4 positive CNV areas was quantified with CellF software (Olympus, Hamburg, Germany).

### Analysis of Retinal and Choroidal Cells by Flow Cytometry

The retina and choroid of the laser-treated eyes were dissected microsurgically 3 and 6 days after laser. The separated tissues were digested in 0.5 mg/ml collagenase and 100 μg/ml DNase I in RPMI 1640 medium (Invitrogen, Karlsruhe, Germany) plus supplements (RPMI^+^ medium). The basical RPMI^+^ medium which was used for incubation of living cells contained 10% heat-inactivated FCS (PAA Laboratories, Pasching, Austria), 1 mM L-glutamine (PAA), 50 μM β-mercaptoethanol and antibiotics (Sigma-Aldrich). For digestion, the choroid was incubated in this digestion medium shaking at 37°C for 40 min, the retina for 30 min. The washed cells were filtered through a 100 μm nylon mesh and incubated in RPMI^+^ for 4 h at 37°C with 0,1% GolgiPlug (BD Biosciences, Heidelberg, Germany) to prevent cellular release of VEGF. Prior to surface staining, Fc receptors were blocked for 20 min on ice using the supernatant of 2.4G2 cells containing the monoclonal 2.4G2 antibody (REF. BD, Cat. 553142) to prevent unspecific binding of antibodies. Cells were stained in PBS lacking Ca^2+^ and Mg^2+^ but containing 0,1% FCS (PAA) and 0,5% sodium azide. For surface staining, titrated amounts of the following labeled monoclonal antibodies from BD Biosciences and BioLegend were used: anti-CD45-PacificBlue (30-F11), anti-Ly6G-V450 (1A8), anti-CD11c-BV605 (N418), anti-CD31-PE (MEC 13.3), anti-F4/80-PE (CI:A3–1), anti-CD11b-alexa488 and -PerCP-Cy5.5 (M1/70). Cells were fixed in 2% PFA (PBS) for 15 min, thereafter permeabilized in PermWash (BD) and 30 min intracellularly stained with the following monoclonal antibodies: For staining, unlabeled anti-RPE65 (ab13826, Abcam), anti-VEGF_120/164_ (39917, R&D Systems) and monoclonal Rat IgG_2b_ (isotype for VEGF-A, eBioscience) were labeled with monoclonal antibody labeling kits from Invitrogen (A-20181 or A-20186), as indicated. Anti-vimentin-PerCP-Cy5.5 (polyclonal, sc-7558, Santa Cruz Biotechnology) was used to identify astrocytes and Müller cells. A defined number of PE- or APC-labeled microbeads (BD Biosciences) were added to calculate the number of cells per sample. Cells were measured on an LSR II cytometer (BD) and analyzed by using Flow Jo software (Tristar). Classification of immune cells was performed as described earlier [4]. To semiquantitatively determine the VEGF content per cell, we determined the mean fluorescence intensity (MFI) after incubation with a VEGF-specific antibody, and subtracted the MFI in a separate sample stained with a non-specific antibody, to eliminate the contribution of non-specific antibody binding to the cell. To obtain a single parameter for the total content of VEGF by a single cell subset, we multiplied this difference with the number of VEGF^+^ cells based on a recently described protocol [24], using the formula: *celltype-specific amount of VEGF* (CA-VEGF)  =  (absolute cell number of Cs) x (MFI_Cs_stained with anti-VEGF_ – MFI_Cs_stained with isotype control_).

### Statistics

Results were analyzed by using GraphPad Prism 5 (GraphPad Software, San Diego, CA) and expressed as mean ± standard deviation (SD). Two-way analysis of variances (two-way ANOVA) was used to evaluate the dependency of MPh numbers after laser injury. Comparisons of cell numbers were drawn using *t* test for two groups or a one-way analysis of variances (one-way ANOVA) in combination with Bonferroni multiple-comparison post test for multiple groups. MFI, CA-VEGF and CNV areas were analyzed by the two-tailed nonparametric Mann-Whitney-U-Test. The p values were expressed as: * <0.05, ** <0.01, *** <0.001.

## Results

### Establishment of a protocol to detect intracellular VEGF in eye cells

**Figure 1:**
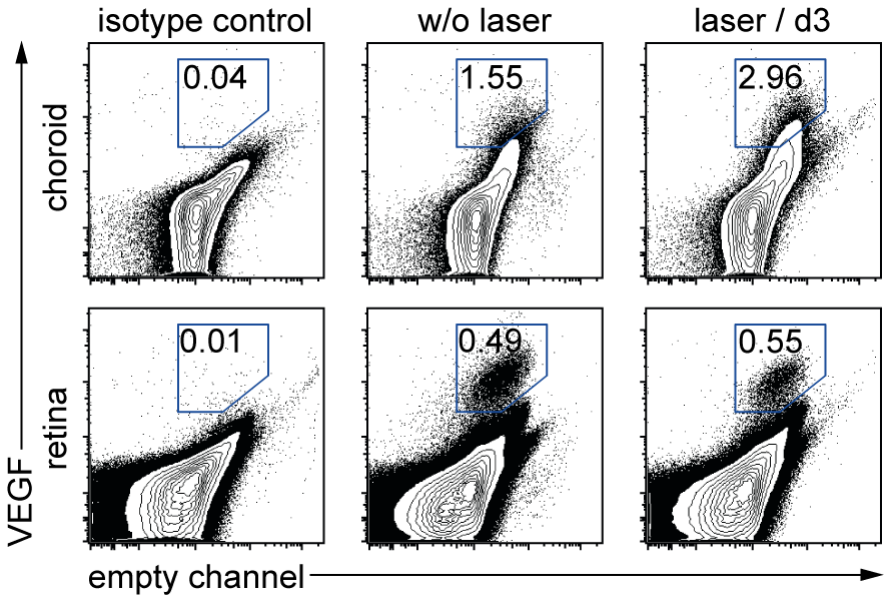
Flow cytometric analysis of VEGF-containing eye cells in the choroid and the retina. VEGF-containing eye cells from the choroid and the retina of untreated (middle) and laser-treated (right) mice are shown. Specificity was verified by an isotype control (left). Cells positive for VEGF were plotted versus an empty fluorescence channel. Percentages are means of the respective group. Dot-plots display representative data from one of two independent experiments. A total of 4–5 mice were used per group in each of these experiments

To identify the cellular source(s) of VEGF in laser-induced neovascularization, we established an intracellular staining protocol followed by flow-cytometric analysis. To this end, single eye cell suspensions were prepared by digesting separated retina and choroid of murine eyes with collagenase. Cells were permeabilized and stained with a fluorochrome-labelled antibody against VEGF or an isotype control. Flow-cytometric analysis revealed autofluorescent cells evident by a fluorescence signal in the empty channel (Fig. 1). In the antibody-stained samples, an additional cell population stained positive for the VEGF-specific antibody both in the choroid and retina, which occupied 1.55±0.15 or 0.49±0.03%, respectively (Fig. 1, middle column). This population was absent in isotype antibody stained controls (Fig. 1 left column), indicating specificity of VEGF staining. We next applied 10 laser spots to the eye fundus to rupture Bruch's membrane and permit CNV growth and analyzed VEGF^+^ cells three days later, when VEGF expression according to published information peaked [10]. Indeed, we found that the VEGF^+^ subset was almost doubled to 2.96±0.44% of all cells in the choroid at this time point, indicating that our protocol allowed reliable flow-cytometric analysis of intracellular VEGF content on the single cell level.

### The VEGF^+^ cell subset contains CD11b^+^ phagocytes, CD11c^+^ dendritic cells, endothelial cells and vim^+^ cells, but not neutrophils

**Figure 2:**
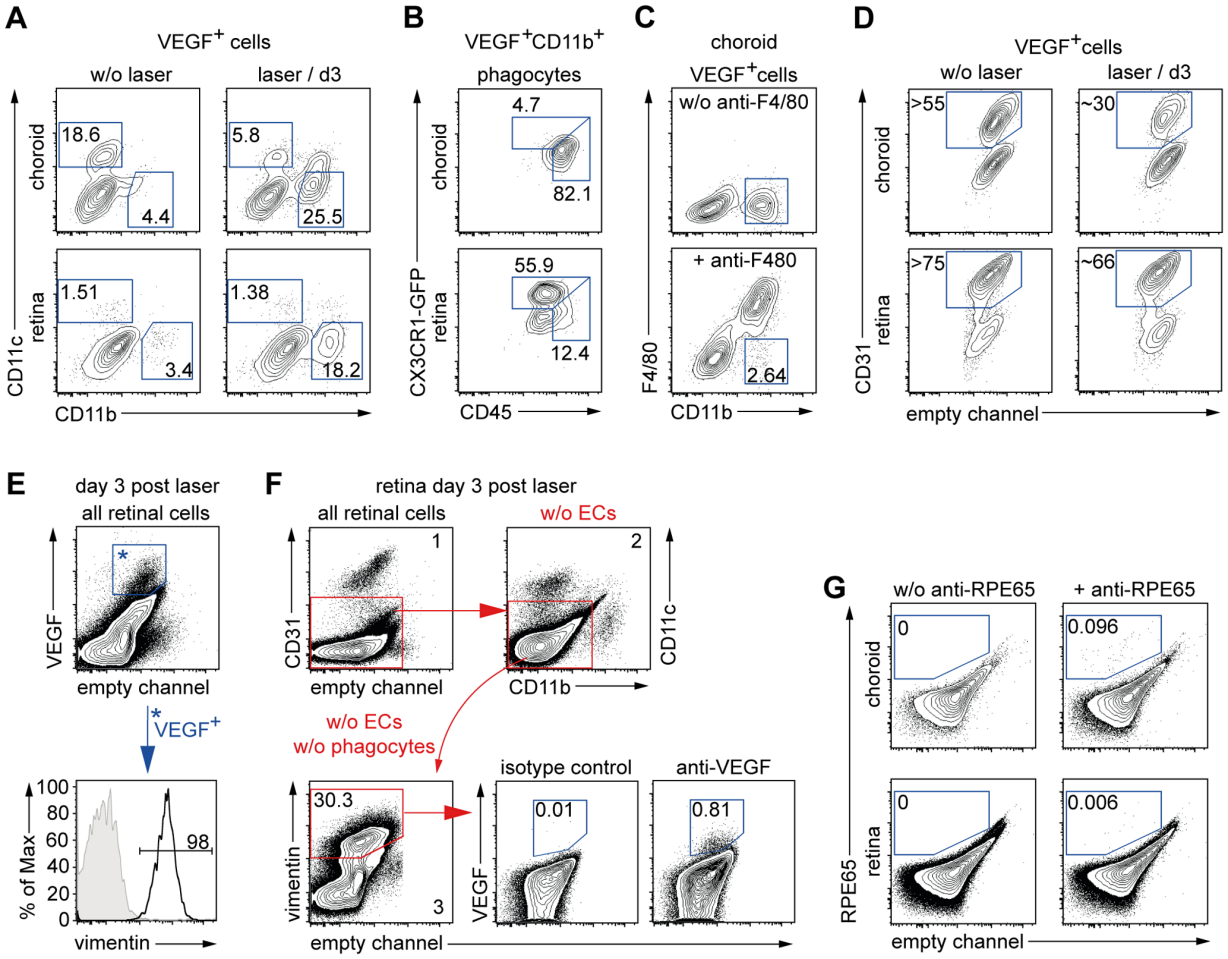
CD11b^+^ mononuclear phagocytes, CD11c^+^ dendritic cells, endothelial cells and retinal vimentin^+^ cells contain VEGF. (A) VEGF^+^ cells were analyzed for CD11b and CD11c expression. CD11b^+^ CD11c^–^ phagocytes and CD11b^–^ CD11c^+^ dendritic cells (DCs) are shown in the choroid and the retina of untreated eyes (left) and 3 days after laser injury (right). (B) The VEGF^+^CD11b^+^ phagocytes were subclassified by analyzing their CD45 and CX_3_CR1 expression at day 3. (C) VEGF^+^CD11b^+^ cells in the lasered choroid were further characterized by analyzing F4/80 expression (bottom), which is present on MP but absent on neutrophils. (D) VEGF^+^ endothelial cells (ECs) were identified by CD31 staining in eyes from healthy mice (left) and after laser at day 3 (right) in the choroid (top) and the retina (bottom). (E) Retinal VEGF^+^ cells were identified as mentioned in Figure 1 and displayed (top). VEGF^+^ cells were stained positive for vimentin (black line in Histogram) versus the control (grey tinted). (F) Gating strategy to exclude CD31^+^ ECs (1) and CD11b^+^ and CD11c^+^ phagocytes (2) from the vim^+^ cell subset of astrocytes and Müller cells (3). This subset was analyzed for VEGF expression compared to the isotype control. (G) RPE cells were stained for the marker RPE65 (right) versus control (left) in the choroid (top) and the retina (bottom). Percentages (A–D) are means of the respective group. Results are representative for two independent experiments. A total of 3–5 mice per group were analyzed in each experiment.

We used our protocol to identify the VEGF^+^ cells by multicolor co-staining with antibodies against cell subset markers (Table 1). Among the immune cells, we focused on CD11b^+^ phagocytes, because previous histological studies by others found these cells to produce VEGF [10]. As a control, we used CD11c to identify DCs. Around 19% of the choroidal VEGF^+^ cells were DCs, but only some cells were CD11b^+^ phagocytes (Fig. 2A). The retina contained few cells of either subset (Fig. 2A). 3 days after laser injury, both compartments contained numerous CD11b^+^ phagocytes but not CD11c^+^ DCs (Fig. 2A), suggesting recruitment from the circulation. When we subclassified the VEGF^+^ CD11b^+^ phagocytes by co-staining for further subset markers, those in the choroid were mostly MPs (CD45^hi^ CX_3_CR1^lo^ F4/80^+^) and those in the retina were mostly microglia cells (μGlia, CD45^l^°CX_3_CR1^hi^) (Fig. 2B). As small subset of VEGF^+^ MPs was present (Fig. 2B).

**Table 1:** Classification of VEGF+ retinal/choroidal cells by intracellular and cell surface markers.

marker	phagocytes				non-immune cells		Staining site
	MP	μGlia	DC	NG	EC	RGC	
**CX_3_CR1**	Int	High	Int	Neg	Neg	Neg	surface
**CD11b**	High	High	Int/Neg	High	Neg	Neg	surface
**F4/80**	High	Int	Int	Neg	Neg	Neg	surface
**CD11c**	Low	Low	High	Neg	Neg	Neg	surface
**CD45**	High	Low	High	High	Neg	Neg	surface
**vimentin**	positive						Intracellular
**CD31**	Neg	Neg	Neg	Neg	Pos.	Neg	surface

[macrophages (MP); microglia cells (μGlia); dendritic cells (DC); neutrophilic granulocytes (NG); endothelial cells (EC); retinal astro/Müller glial cells (RGC); intermediate (Int); negative (Neg)].

Neutrophilic granulocytes (NG, CD45^+^ CD11b^+^ F4/80^−^) were absent (Fig. 2C), confirming that VEGF^+^ CD11b^+^ cells were mononuclear phagocytes like MPs or microglia cells. We also detected numerous CD31^+^ endothelial cells among the VEGF^+^ cells in retina and choroid (Fig. 2D). In the untreated choroid or the retina, ECs represented more than 55% or 75% of all VEGF^+^ cells. In the choroid, they constituted about 30%, and in the retina ∼66% of VEGF^+^ cells upon laser injury (Fig. 2D). Among the retinal non-immune cells reported to produce VEGF are astrocytes [18], [19] and Müller cells [20], [21]. We used vimentin (vim) to identify these cells [25]–[27], because GFAP staining [28] in our hands gave inferior staining results for this cell type using flow-cytometry (data not shown). All VEGF^+^ cells of the retina were vim^+^ (Fig. 2E), implying that also the VEGF producing MPs and ECs must be vim^+^. Also ganglion cells have been reported to produce VEGF [29]. As histological data of other groups suggest neuronal cells to be negative for vim [30], our finding that all VEGF^+^ cells were vim^+^ (Fig. 2E) argues against VEGF production by ganglion cells.

To examine astrocytes and Müller cells, we excluded ECs and phagocytes from analysis of the retinal cells by the gating strategy shown in Fig. 2F. and then analyzed the remaining vim^+^ retinal cells for VEGF expression. Only a small subset (∼0.81%) of these cells stained positive for VEGF (Fig. 2F), arguing against a major role for VEGF production by these cells.

Finally, we examined RPE cells, which have been reported to produce VEGF as well [22]. However, we hardly detected any cells positive for the marker RPE65 (Fig. 2E), preventing further analysis of these cells. In summary, ECs, MP, microglia, dendritic cells, endothelial cells and vim^+^ cells (astrocytes/Müller cells) produced VEGF after laser, but neutrophils did not, and RPE cells were not analysable by flow-cytometry.

### Only CD11b^+^ phagocytes but neither endothelial cells, dendritic cells nor vim^+^ cells increase VEGF-production after laser injury

**Figure 3:**
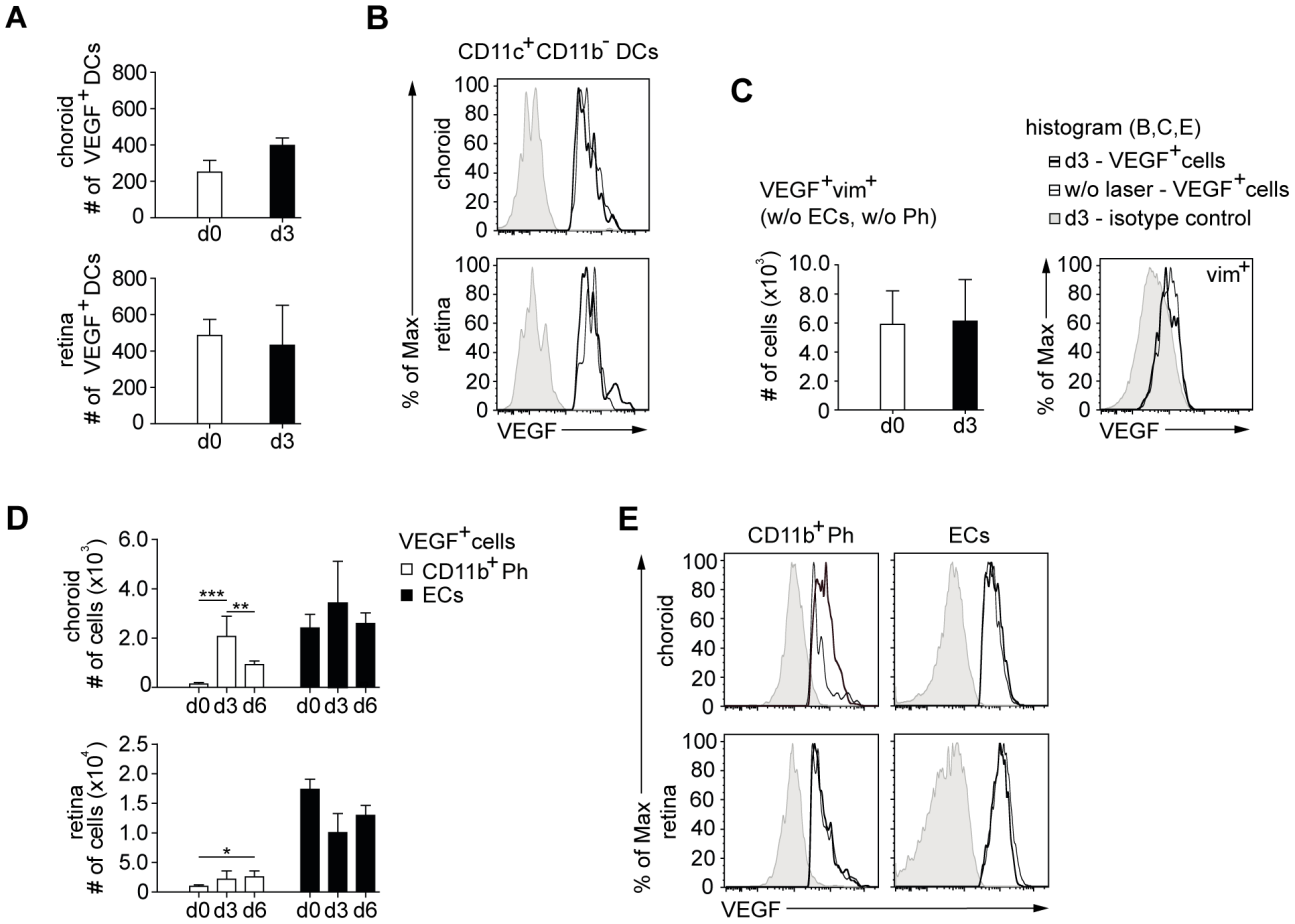
Higher VEGF content in CD11b^+^ phagocytes but neither in other phagocytes nor non-immune cells after laser injury. (A) VEGF^+^ CD11b^−^ CD11c^+^ DCs were enumerated in the choroid (top) and the retina (bottom). (B) VEGF content of these DCs without and after laser treatment (histogram). (C) Number of VEGF^+^ astrocytes and Müller cells (top) and their content of VEGF (histograms). CD31^+^ ECs and CD11b^+^ CD11c^+^ phagocytes were excluded as shown in Fig. 2F). (D) Analysis of cell numbers for VEGF^+^ ECs and CD11b^+^ phagocytes of the choroid and the retina in untreated eyes and 3 and 6 days after laser injury. (E) VEGF-expression of CD11b^+^ phagocytes and ECs without and after laser treatment in the choroid and the retina. Histograms show VEGF content for cell subsets of untreated eyes (small black line) and 3 days after laser injury (thick black line). The isotype control for the respective cell type in lasered eyes (grey tinted) depicts the background fluorescence. Bar graphs show means ± SD. Comparisons of cell numbers (D) were performed using one-way analysis of variances (one-way ANOVA) in combination with Bonferroni multiple-comparison post-test. The statistical significance was analyzed as indicated (* <0.05, ** <0.01, *** <0.001). A total of 3–5 mice per group were analyzed in 2 independent experiments.

We reasoned that laser-induced neovascularization can only result from those cells that upregulate VEGF from the steady-state situation. Therefore, we decided to determine which of the cells identified above upregulated VEGF production in response to laser injury. We examined both the numbers of the VEGF^+^ cells and their mean VEGF content on a per cell basis (MFI for VEGF). Numbers of VEGF^+^ DCs increased slightly in the choroid but not in the retina on day 3 after laser injury (Fig. 3A) and VEGF content per DC was unchanged (Fig. 3B), arguing against a role of DCs. Despite the slight increase of VEGF^+^ DC numbers in the choroid, there was a decrease of the proportion of VEGF^+^ cells (Fig. 2A), because the increase of macrophage numbers was far greater (Fig. 3D). Thus, DCs constituted a smaller proportion among the recruited phagocytes. The numbers and expression of VEGF by vim^+^ astrocytes and Müller cells were unchanged after laser injury (Fig. 3C). However, the number of VEGF^+^ MPs increased more than 15fold in the choroid on day 3 (139±60 to 2066±822 cells), but had already declined to 923±145 cells on day 6 after laser on these days (Fig. 3D). In the retina, microglia cell numbers increased only 2.2fold from 981±236 to 2180±1402 on day 3, but interestingly there was no decline on day 6 (Fig. 3D). The VEGF content per MP was increased on day 3 after laser, whereas microglia cells showed unchanged VEGF levels per cell (Fig. 3E).

The number of choroidal ECs containing VEGF was somewhat increased on day 3, but this was not significant, and it was not detectable on day 6 after laser (Fig. 3D). Numbers of retinal VEGF^+^ ECs were mostly decreased at day3 after laser (Fig. 3D), perhaps reflecting the destruction of EC due to laser treatment. The content of VEGF per EC was not increased in either compartment (Fig. 3E).

In conclusion, among the VEGF^+^ cells in the eye, only choroidal MPs increased their VEGF content after laser injury. Furthermore, a higher proportion of choroidal MPs and retinal microglia cells contained VEGF after laser injury, suggesting that these cell types contribute to CNV.

### Laser-induced VEGF upregulation by choroidal MPs, but not by retinal microglia cells is CCR2-dependent

**Figure 4:**
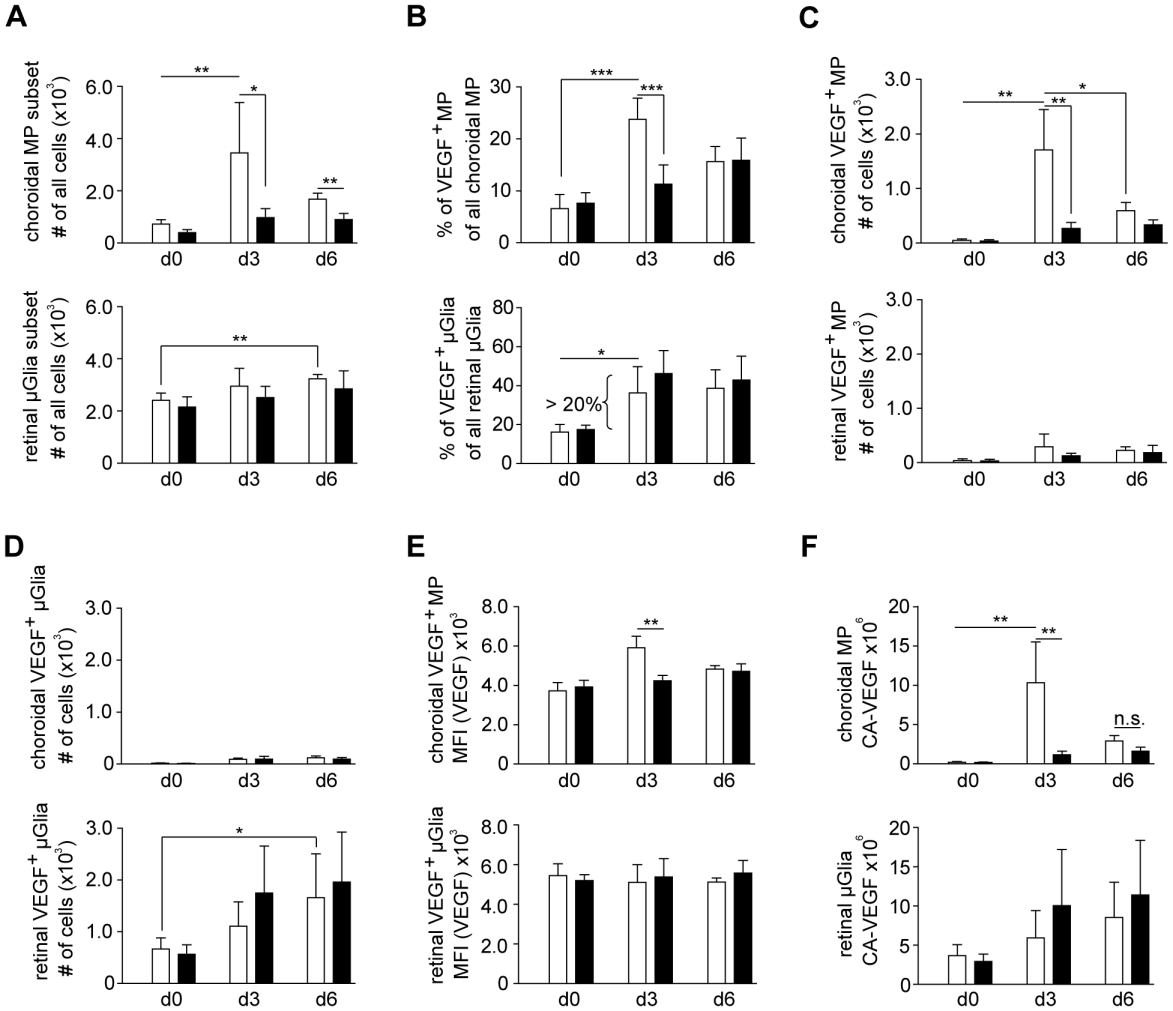
Laser-induced VEGF content by choroidal MPs, but not by retinal microglia cells is CCR2-dpendent. (A) Total numbers of choroidal macrophages (MP) (CD45^hi^ CX_3_CR1^lo^) and microglia cell (μGlia) (CD45^l^°CX_3_CR1^hi^) in CCR2-competent (white bars) or –deficient (black bars) mice before or 3 or 6 days after laser treatment. (B) The percentage of VEGF^+^ cells in the MP and μGlia subset were. (C, D) numbers of VEGF^+^ choroidal MP (C) and (D) VEGF^+^ retinal μGlia. (E, F) VEGF-expression as mean fluorescence intensity (MFI) (E) and calculated amount of VEGF (CA-VEGF) per choroidal MP and retinal μGlia (MFI) (F). Bar graphs show means ± SD. Comparisons of cell numbers were performed using *t* test (A–D). MFI and CA-VEGF were analyzed by the two-tailed nonparametric Mann-Whitney-U-Test (E,F). The statistical significance was analyzed as indicated (* <0.05, ** <0.01, *** <0.001). Two-way analysis of variances (two-way ANOVA) depicted significant differences (***p < 0.001) for MP but not μGlia numbers in comparison of CCR2-competent and deficient reporter mice. A total of 3–5 mice per group were analyzed in 2 independent experiments.

Inflammatory monocytes, which can give rise to MPs, usually depend on CCR2 [13], [14], [31], and this has been observed also for MPs in the eye [17]. When we lasered CCR2-deficient mice, 3fold lower total MP numbers were seen in the choroid after 3 days compared to wildtype mice (Fig. 4A). At 6 days after laser injury, the difference in MP numbers between CCR2-competent and –deficient mice was less substantial, because MP numbers in the former, but not in the latter mice had decreased (Fig. 4A, upper panel). We also examined microglia cells as the most abundant VEGF^+^ myeloid cell subset in the retina. As previously described [4], a slight increase of these retina-resident cells was seen on day 6, but this was CCR2-independent (Fig. 4A, lower panel).

We next focused on those MPs that contained more VEGF after laser injury. The increase in the choroid of their proportions among all MPs (Fig. 4B) and of their absolute numbers (Fig. 4C) was mostly CCR2-dependent. On day 6, much of this accumulation had ceased in CCR2-competent mice (Fig. 4C). Also the retina contained VEGF^+^ MPs, but their numbers were much lower than in the choroid (Fig. 4C). The proportion (Fig. 4B) and numbers (Fig. 4D) of VEGF^+^ microglia cells was increased in the retina on days 3 and 6 (Fig. 4B), and this must be due to induction of VEGF in a higher proportion of microglia cells, because the absolute numbers of microglia cells increased only slightly (Fig. 4A). This increase was CCR2-independent (Fig. 4B+D), coinciding with the fact that microglia cells do not express CCR2 [32]. The choroid contained hardly any VEGF^+^ microglia cells (Fig. 4D).

When we determined VEGF content per cell, choroidal MPs contained significantly more VEGF per cell on day 3 after laser, and this was CCR2-dependent (Fig. 4E), suggesting increased VEGF production by inflammatory macrophages. The VEGF^+^ microglia cells in the retina did not upregulate their VEGF content per cell (Fig. 4E).

### VEGF content per cell type after laser injury

The contribution of a given cell type to intraocular VEGF production depends on the numbers of VEGF^+^ cells of that type and by the average VEGF-content per cell. To obtain a single parameter for VEGF content, we calculated the VEGF production per cell (cell type-specific amount of VEGF, CA-VEGF). To this end, we subtracted from the MFI of the VEGF signal the MFI after staining with an isotype control antibody, in order to remove the non-specific component of the fluorescence signal. Then we multiplied this difference with the number of VEGF^+^ cells.

The CA-VEGF of choroidal MPs increased from 0.19×10^6^ to about 10.31×10^6^ (54fold) on day 3 and was reduced to 2.88×10^6^ on day 6 (Fig. 4F). This increase was dependent on CCR2, as CCR2-deficient mice only showed a 6-7fold increase to 1.14×10^6^ at day 3 (Fig. 4F). The CA-VEGF of retinal MPs was very small (data not shown), due to their low numbers (Fig. 4F). The CA-VEGF of retinal microglia cells increased from 3.7×10^6^ in healthy mice to 5.93×10^6^ on d3 and to 8.53×10^6^ on day 6, whereas no significant changes were noted in CCR2-deficient mice (Fig. 4F). Taken together, these findings indicated that laser treatment increased VEGF content by resident μGlia cells and temporarily through CCR2-dependent recruitment of MPs into the choroid.

### CCR2-dependent MPs transiently increase choroidal neovascularization

**Figure 5:**
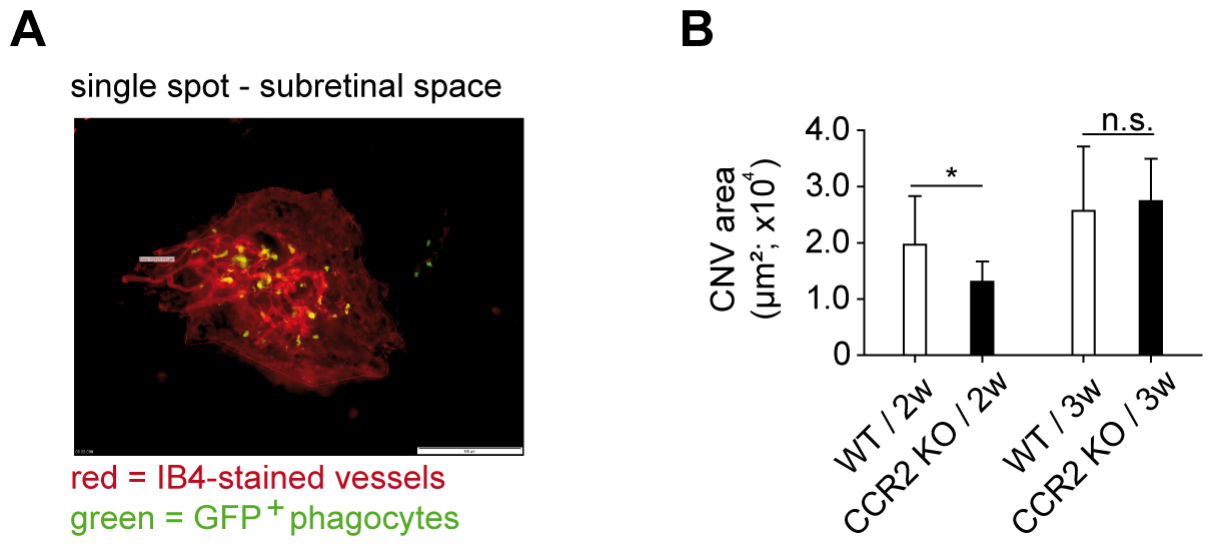
CCR2-deficiency temporarily reduced CNV areas in choroidal flatmounts. (A) Fluorescence microscopy of Isolectin B4-stained choroidal flatmounts of eyes from CX_3_CR1-GFP reporter mice showing the endothelium in neovascular areas with GFP^+^ phagocytes (single spot; scale bar = 100 μm). (B) Isolectin B4 (IB4)-positive CNV areas from CX_3_CR1 reporter mice lacking (black bar) or expressing CCR2 (black bar) after 2 (2w) or 3 weeks (3w) following laser treatment. Bar graphs show means ± SD. A total of 3–5 mice were used per group 2 independent experiments. CNV areas were analyzed by the two-tailed nonparametric Mann-Whitney-U-Test. The statistical significance was analyzed as indicated (*p<0.05, n.s.  =  not significant).

We finally asked whether the CCR2-dependent influx of VEGF^+^ MPs had consequences for choroidal neovascularization. To this end, we lasered CX_3_CR1 reporter mice (CX_3_CR1^GFP/+^), which allow direct visualization of MPs, and examined flatmounts of their choroid after 2 weeks. When we revealed neovascular areas by staining with the endothelial marker, isolectin B4 (IB4), GFP^+^ MPh colocalized with these vessels (Fig. 5A). The lack of CCR2 caused a significant reduction of the CNV two weeks after laser treatment (Fig. 5B), consistent with previous reports [17]. However, given that GFP^+^ phagocytes are detectable in laser spots longer than 2 weeks after laser injury, and given that our analysis above revealed an increased VEGF content by CCR2-independent cells beyond early time points, we decided to examine a later time point as well. Indeed, three weeks after laser, no significant differences between the extents of neovascular areas in CCR2-competent and -deficient animals were evident any longer (Fig. 5B). This indicates that the transient increase of VEGF by CCR2-dependent influx of MPs augmented neovascularisation only temporarily, presumably because the VEGF production by CCR2-independent cells prevailed at later time points.

## Discussion

The intraocular production of VEGF is critical for neovascularisation in human AMD and in laser-induced CNV, which often serves as a murine model of AMD. The present study to our knowledge is the first to employ flow-cytometry to identify the cellular sources of VEGF in this model. A major advantage of flow-cytometry is the ability to perform multicolor analysis, which allows the concurrent use of several additional markers to identify VEGF^+^ cells. This allowed demonstrating that ocular endothelial cells, vimentin^+^ cells (astrocytes or Müller cells), and several myeloid immune cells, namely MPs, DCs and microglia cells, but not neutrophil granulocytes, contained VEGF.

VEGF-content in the steady state was highest in endothelial cells, and in the retina also in microglia cells. RPE cells have been previously described to produce VEGF [22], but were not identifiable by flow cytometry, which might be due to their loss during cell isolation or due to physical properties like the dark pigmentation of these cells. Some cells were not classifiable with our technique, and may include for example fibroblasts, which have been shown to produce VEGF in vitro after stimulation with pro-inflammatory cytokines [33].

Three days after laser injury, only macrophages contained substantially increased VEGF levels, which was due both to increased numbers of VEGF^+^ macrophages and by higher VEGF content per macrophage. By combining these two parameters mathematically, we obtained the CA-VEGF as a parameter for the VEGF-content in a cell subset. 3 days after laser, MPs constituted around 80% of VEGF^+^ phagocytes in the choroid and CA-VEGF in the choroid was 54fold increased. Although macrophages constituted a comparatively small subset among the VEGF^+^ cells, their contribution to CNV may nevertheless be relevant, because also the fine location of the VEGF-producing cells is important. We and others had previously shown that myeloid immune cells are preferentially recruited to the laser spots [4], [17], and might secrete VEGF exactly were new blood vessels are needed.

The CA-VEGF of microglia cells was also increased, albeit only by a small amount, and our analysis revealed that this was due to a larger subset of the microglia cells that began producing VEGF, rather than to higher production per cell. The VEGF production also of these cells may also be focused to sites of injury, and may substantially contribute to the local generation of blood vessels. Indeed, microglia cells have been reported to translocate from the inner retina to the subretinal space both in laser-induced CNV and in humans with AMD [5]. Given that these cells produce VEGF, this migration may be pathophysiologically relevant. Also the longer duration of VEGF production argues for a pathogenic role of microglia cells.

Like microglia cells, also the CD11c^+^ DCs of the eye failed to upregulate VEGF per cell, but their motility might allow them to relocate towards laser spots. Others have shown that intravenously injected immature DCs accumulated in CNV lesions and enhanced their size [34]. However, despite their principal ability to influence CNV, their small numbers argue against a substantial increase of overall ocular VEGF production. They might, however, affect CNV by other mechanisms.

We failed to detect VEGF within neutrophils in the choroid. Nevertheless, neutrophils might indirectly affect CNV by stimulating other VEGF-producing cells. This may explain previous studies showing that neutrophil depletion by antibodies reduced CNV in mice under certain conditions [35].

Intrinsic eye cells, at least those we could analyze, like astrocytes or Müller cells, and other non-immune cells that we could not further classify, did not increase their VEGF content in response to laser treatment and are not motile.

The rapid accumulation of VEGF^+^ MP can theoretically be explained by the recruitment of circulating precursors, i.e. inflammatory monocytes, and this is supported by a previous study showing that their recruitment on day 3 was correlated with increased VEGF levels in laser-induced CNV [10]. As inflammatory monocytes usually depend on CCR2, we analyzed cellular VEGF content in CCR2-deficient mice. Indeed, the transient accumulation of VEGF^+^ choroidal MP on day 3 was entirely CCR2-dependent. This is consistent with a previous study reporting that VEGF in flow cytometry-sorted eye macrophages after laser injury was reduced after pharmacological CCR2 blockade [Xie, 2011). In contrast, the lack of CCR2 reduced neither the number of VEGF^+^ microglia cells nor their VEGF content, at least at the time points investigated here. This is consistent with previous studies concluding that microglia cells are derived from long-term resident microglia cells, rather than from circulating precursors recruited to the eye [36] and that these cells lack CCR2 expression [32]. Interestingly, CCR2-deficient mice showed a small but reproducible increase in numbers of VEGF^+^ MPs. This may indicate that these cells may use another mechanism to enter the eye, or that they might be derived from a different, CCR2-independent precursor. A likely candidate are CX_3_CR1-dependent “patrolling monocytes”, a distinct monocyte subset that surveys blood vessels and can enter sites of inflammation [37]. There is evidence that ocular MPs are CX_3_CR1-dependent in some models [38]-[40]. Future studies are required to clarify whether the CCR2-independent VEGF-producing MPs are CX_3_CR1-dependent.

The intracellular detection of VEGF may indicate cell-intrinsic production or uptake of VEGF from other sources after binding to VEGF receptors on the cell surface. ECs belong to the prime target cells of VEGF; they express particularly strong levels of VEGF receptors, and can use them to internalize this cytokine [41]. Consequently, the VEGF that we detected within ECs might not or only partially be produced by the ECs themselves. Macrophages express lower levels of VEGF receptors [42], and therefore should be less capable of capturing exogenous VEGF. On the other hand, numerous publications have established macrophages as potent producers of VEGF by analyzing their VEGF mRNA content [43]–[45]. Especially in tumors, a role of macrophage-derived VEGF was suggested [46], [47]. VEGF supports tumor angiogenesis and leads to formation of vessels that are similarly irregular and leaky as those in CNV [48], [49]. Furthermore, our protocol of intracellular VEGF staining used Brefeldin A, which prevents intracellularly synthesized molecules from being secreted, causing them to accumulate intracellularly. Thus, the accumulation of intracellular VEGF after Brefeldin A treatment supports intracellular production. Finally, we saw reduced CNV in CCR2 KO mice lacking inflammatory macrophages, consistent with observations by others reporting that flow-cytometry sorted macrophages increased their content of VEGF and VEGF mRNA after laser-induced CNV, unless a CCR2 antagonist was applied [16]. If macrophages had scavenged VEGF produced by other cells, rather than producing it themselves, then more rather than less free VEGF should be available in their absence, and CNV in CCR2 KO mice should have been higher. The opposite was the case in our study and in studies by others [10], strongly supporting the production of VEGF by macrophages.

In conclusion, our findings support the conclusion that the early laser-induced increase in VEGF production in the choroid is due to CCR2-dependent MPs, and that CCR2-independent cells, such as microglia cells play a larger role at later time points. This would predict a transient reduction of CNV in the absence of CCR2, and this is indeed what we observed, as the difference in CNV after 2 weeks was not observable any longer after 3 weeks. This argues against CCR2 as a useful therapeutic target in AMD. However, it has to be kept in mind that laser-induced CNV is initiated by one injurious event, whereas human AMD likely entails prolonged inflammatory signals that might continuously recruit VEGF-producing MPs. Studies using our technique to determine VEGF in models of chronic ocular inflammation are warranted to clarify the therapeutic value of CCR2-inhibition in AMD.
